# Evaluating Self-Directed Rehabilitation for Knee and Hip Arthroplasty During the COVID-19 Pandemic: A Multicenter Study

**DOI:** 10.3390/medsci12040069

**Published:** 2024-11-26

**Authors:** Todd M. Miner, Mike B. Anderson, David C. Van Andel, Robert E. Neher, Roberta E. Redfern, Paul J. Duwelius

**Affiliations:** 1 Colorado Joint Replacement, Denver, CO 80210, USA; todd.miner@adventhealth.com; 2 Zimmer Biomet, Warsaw, IN 46580, USA; mike.anderson@zimmerbiomet.com (M.B.A.); robert.neher@zimmerbiomet.com (R.E.N.); 3 Orthopedic and Fracture Specialists, Portland, OR 97225, USA; pduwelius@gmail.com

**Keywords:** knee arthroplasty, hip arthroplasty, mobile health, rehabilitation, COVID-19

## Abstract

The COVID-19 pandemic has triggered the adoption of new technologies to reduce the need for in-person physical therapy (PT). This study evaluated the impact of the COVID-19 pandemic on PT utilization and outcomes of patients prescribed a smartphone-based care management platform (sbCMP) for self-directed rehabilitation (SDR). A secondary analysis of data collected in a multicenter, prospective cohort trial investigating a mobile platform to deliver SDR after arthroplasty was performed. Patients who used the sbCMP for 2 weeks prior to undergoing partial knee arthroplasty (PKA), total knee arthroplasty (TKA), or total hip arthroplasty (THA) and provided 3 months of post-operative data were included. Use of adjunct PT at 3 months, step counts, and KOOS JR and HOOS JR scores were compared during the early versus late pandemic period. The cohort data was available for 1665 patients. Use of SDR without adjunct PT was higher in the early period of the COVID-19 pandemic in TKA (35.3% vs. 27.6%, *p* = 0.03) and THA (72.5% vs. 59.3%, *p* < 0.001), but not in the PKA cohort (58.9% vs. 53.3%, *p* = 0.53). Post-operative step counts improved at 3 months compared to pre-operative levels in all procedure types. Change in HOOS JR and KOOS JR scores from pre-operative to post-operative levels were similar by pandemic period in all cohorts. Use of SDR increased early during the COVID-19 pandemic, corresponding to pandemic restrictions, without negatively impacting patient outcomes. SDR facilitated via a sbCMP may be beneficial for patients unable or unwilling to participate in traditional PT visits.

## 1. Introduction

The COVID-19 pandemic resulted in restrictions to healthcare services deemed non-urgent, particularly during the early phases when lockdowns were common [1]. These restrictions had a particularly strong impact in the field of physical therapy, where rehabilitation was often considered a non-urgent health service across many disciplines [2,3]. During the early phases of the COVID-19 pandemic, many healthcare providers realized the need to quickly pivot to the use of technology to provide continuity of care to their patients, as patients’ access to services such as physical therapy were highly restricted. In the United States, payors recognized the need to adjust service models, where barriers to billing for telerehabilitation were removed by Centers for Medicare and Medicaid (CMS) and commercial insurers alike [4,5]. Many centers described their experience and success in implementing videoconferencing, telehealth, and other patient monitoring methods. However, most of these centers focused on patient and clinician acceptance and satisfaction with newer technology rather than clinical effectiveness of the approach [6,7,8,9,10]. The need for “off-the-shelf” options that are simple to implement and do not require patients and clinicians to acquire sophisticated equipment was also asserted [11]. Given the notable advantages of telerehabilitation, specifically the potential for reduction in resource utilization by healthcare systems, authors recommended further investigation and utilization beyond the COVID-19 pandemic [12,13], as the need for physical therapy is expected to continue to increase as demand for total joint arthroplasty procedures is anticipated to grow [14].

This medium of delivering physical therapy has demonstrated non-inferior outcomes with potential cost savings compared to in-person physical therapy programs [15,16], with many authors reporting high levels of patient satisfaction and adherence to therapy. This is particularly true in studies of telerehabilitation in patients after arthroplasty, where outcomes have been equal to, or in some cases, superior to in-person physical therapy following joint replacement [15,17,18]. Though much research has begun to focus on the ability of telerehabilitation to provide equivalent results post-operatively, few studies have detailed the effectiveness of mobile applications to guide self-directed rehabilitation (SDR) following arthroplasty [19,20,21,22]. Moreover, there is a paucity of evidence relating to the effectiveness of the adoption of mobile applications. This study aimed to investigate the impact of the COVID-19 pandemic on adjunct physical therapy use in patients prescribed a mobile application for SDR following arthroplasty procedures. In addition, we evaluated objective (step counts) and subjective (patient reported outcome measures, PROMs) recovery to determine whether outcomes were impacted by changes in access to in-person physical therapy.

## 2. Methods

A secondary analysis of data collected in a multicenter, prospective cohort trial was performed (ClinicalTrials.gov #NCT03737149). Enrolled patients deemed appropriate for remote care were prescribed the use of the smartphone-based care management platform (sbCMP) mymobility^®^ (Zimmer Biomet, Warsaw, IN, USA), an iOS (Apple, Inc., Cupertino, CA, USA) and Android-supported (Google, LLC, Mountain View, CA, USA) application that provides pre- and post-operative education and collects PROMs corresponding to the user’s progression through the episode of care, as previously described [19,20]. Daily physical therapy exercise videos, specific to the procedure and point in the episode of care, are provided by the application with reminders to complete assignments, facilitating self-directed rehabilitation. For example, knee arthroplasty cases can be assigned exercises such as manual knee extension, seated kicking, seated heel slide, seated quad set, seated knee extension, seated marching, heel pulls, and ankle pumps, among others, every morning and evening. Appropriate exercises are assigned to hip cases, including some of the aforementioned exercises as well as leg sideways slide, hooklying clamshell, straight leg raise with stabilization, and several others. Clinicians have the ability to view compliance with exercises within the care team-facing dashboards. The sbCMP includes Apple Watch^®^ connectivity to passively collect activity data, including daily step counts. Enrollment criteria include an age of 18 years or older, ownership of an iPhone capable of pairing with the Apple Watch, and pre-operative mobility requiring no more than a single cane or crutch. Patients known to have alcohol or substance abuse, inflammatory arthropathies, or planning bilateral replacement either simultaneously or staged <90 days apart were excluded from participation. All subjects were scheduled to undergo total knee arthroplasty (TKA), partial knee arthroplasty (PKA), or total hip arthroplasty (THA) at the time of enrollment and were required to download the application at least two weeks prior to their scheduled surgery date. Only those who provided at least three months of post-operative data were included in this analysis. Subjects were treated according to each institution’s standard of care, including adjunct outpatient physical therapy where applicable. The study was approved by the Institutional Review Board prior to commencement; written informed consent was provided by each subject at enrollment.

**Figure 1:**
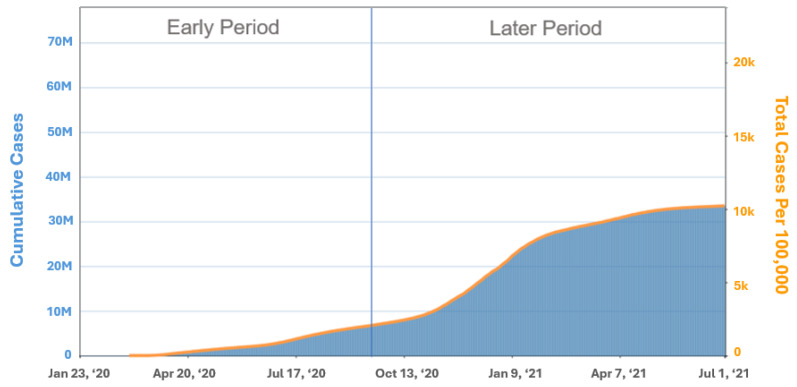
Study definition of early and late period of the COVID-19 pandemic. Trends in Total Cases and Cumulative Incidence Rate of COVID-19 Cases in the United States Reported to CDC, per 100,000 population (https://covid.cdc.gov/covid-data-tracker/#trends_totalcases Accessed on 4 September 2021) are available on the agency site at no charge. Use of CDC materials in this publication does not constitute endorsement or recommendation by the U.S. Government, Department of Health and Human Services, or Centers for Disease Control and Prevention.

Only patients whose surgeries occurred between 1 January 2020 and 30 June 2021 were included. Subjects were separated into two groups according to their date of surgery respective to phases of the COVID-19 pandemic. From 1 January 2020–30 September 2020 was considered the early pandemic period, to include the date of initial emergence of the disease in the United States, implementation of screening protocols, and public awareness and fear of disease as well as implementation of lockdowns and restrictions (Figure 1) [23]. These were compared to cases that occurred later in the pandemic (1 October 2020–30 June 2021) in which restrictions were eased and access to non-urgent healthcare services was considered more available, though patient acceptance of non-urgent services may have been reduced given the high levels of COVID-19 transmission that occurred during this time according to the Centers for Disease Control and Prevention tracker for the United States. Use of adjunct PT following arthroplasty was compared by pandemic period, as were objective (step counts) and subjective outcomes (PROMs) to quantify the effects of the SDR guided by the sbCMP during the COVID-19 pandemic.

Baseline patient demographics were collected at the time of enrollment including sex, age, and body mass index (BMI, kg/m^2^). Step counts were passively collected throughout the study period: two-day mean step counts in the pre-operative timeframe and at 90 days post-operative. Step counts were also investigated by procedure type and COVID-19 period. Patients undergoing knee and hip arthroplasty completed validated PROMs (KOOS JR or HOOS JR) respective of their scheduled procedure types pre-operatively and at 90 days following surgical intervention. At 90 days following their procedure, patients self-reported the use of adjunct PT during recovery. This timeframe was chosen as it represents the Centers for Medicare and Medicaid definition of the episode care for hip and knee arthroplasty procedures.

### Statistical Analysis

Descriptive statistics are reported as mean ± SD, while categorical data are presented as counts and frequency. Continuous data were compared by Student’s *t* test where normally distributed, or by Mann–Whitney U test when Shapiro–Wilk test of normality indicated non-parametric distribution. Categorical data were compared by period using Fisher’s Exact test. PROMs were investigated by change in KOOS JR and HOOS JR score from pre-operative levels to those reported at 90 days as a function of procedure type and pandemic period. All statistical analyses were performed using SAS Version 9.4 (2013, SAS Institute, Inc., Cary, NC, USA). Two-tailed *p*-values < 0.05 were considered statistically significant.

## 3. Results

During the study period, 1665 enrolled subjects underwent lower limb arthroplasty, 766 (46.6%) had TKA, 193 (11.6%) underwent PKA, and 706 (42.4%) were THA cases. On average, the entire cohort was 63.1 ± 9.5 years of age, and the majority (55.7%) were female. In total, 551 patients were included who underwent arthroplasty during the early phase of the pandemic, with 1114 receiving surgical intervention in the later period. Baseline patient characteristics among each procedure type were similar between those separated into early and late pandemic periods as shown in Table 1, though more females underwent THA in the early period.

**Figure 2:**
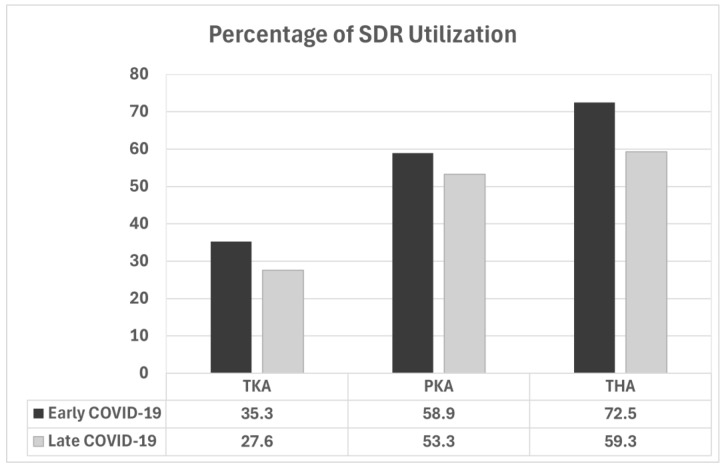
Percentages of patients utilizing only SDR as guided by the sbCMP by procedure and period of the pandemic (early, March–September 2020; late, October 2020–July 2021).

Overall, 878 (52.7%) of the total cohort reported treatment by a physical therapist for rehabilitation for the joint replaced during their index procedure, which occurred less frequently in the early phase of the pandemic (46.8% vs. 55.7%, *p* < 0.001), corresponding with associated lockdowns and care restrictions. However, the effect size was small (φ = 0.08). The use of SDR without adjunct PT was higher in the early COVID-19 period across all procedure types (Figure 2) but did not reach statistical significance in those who had PKA (58.9% vs. 53.3%, *p* = 0.53). In the TKA cohort, significantly more patients used SDR alone in the early COVID-19 period (35.3% vs. 27.6, *p* = 0.03, φ = 0.08). Similarly, significantly more THA patients used SDR without adjunct PT in the early period (72.5% vs. 59.3%, *p* = 0.0007, φ = 0.13) however the effect sizes (φ) are small.

Step counts as collected by participants’ wearables were recovered and exceeded pre-operative levels in all procedure types, regardless of pandemic period (Table 2). Mean pre-operative step counts were compared by t test between those in the early and late pandemic, according to procedure type, and did not vary significantly between the two periods (Table 2). Similarly, post-operative step counts were similar between periods across all procedure types. Those undergoing PKA performed on average more steps than did the TKA or THA cohorts, both pre-operatively and post-operatively.

Examination of PROMs from pre-operative to 90 days post-operative also demonstrated significant improvement irrespective of pandemic period. KOOS JR scores improved by 18.2 points in those undergoing TKA, 16.4 points in patients who had PKA, and HOOS JR scores increased 28.9 points on average in the THA cohort. Considering the pandemic period in which the procedure took place, there was no difference in the change in PROMs scores in any procedure type (Table 3), TKA (17.7 vs. 18.4, *p* = 0.54), PKA (16.0 vs. 16.5, *p* = 0.80), and THA (28.6 vs. 29.0, *p* = 0.75). Of note, the incidence of manipulation under anesthesia was not higher in the cohort using SDR alone compared to those with increased use of adjunct PT (2.8% vs. 4.2%, *p* = 0.37).

## 4. Discussion

We have demonstrated that SDR guided by a sbCMP was utilized without adjunct physical therapy after arthroplasty in patients enrolled in this study more frequently in the early period of the pandemic, when restricted access was common. Importantly, both objective and subjective measures of recovery, including step counts, KOOS JR, and HOOS JR scores did not vary according to the period of the pandemic, suggesting that SDR provided by the sbCMP can be feasibly and effectively utilized, particularly when access to in-person physical therapy is hindered.

The use of telehealth solutions to deliver healthcare increased substantially during the COVID-19 pandemic [5]. However, as our data shows, even in the early pandemic period, access to traditional PT was not completely interrupted. While a larger proportion of patients relied on SDR following arthroplasty in the early phase of the COVID-19 pandemic, when stay-at-home orders were in place for much of the United States [1], nearly half of our participants sought adjunct PT during this time. This may support reports in the literature that suggest that many patients prefer in-person care as opposed to telerehabilitation. Alternately, the observed use of PT in this cohort may be due to clinician preferences or institutional standards of care. Dierick et al. suggested that clinical staff felt that telerehabilitation was a good option when in-person therapy was not available, but most did not agree that it could totally replace in-person PT [24]. Similarly, others suggest the use of telerehabilitation as a supplement rather than a substitution for in-person delivery of PT [7]. This may be supported by the fact that early period SDR-only use in this study was statistically significant, but the effect sizes were small. In previous RCTs of this sbCMP, patients who were randomized to use the mobile application were not immediately prescribed adjunct PT according to institutional standards, receiving those services only in cases where gait difficulties, range of motion deficits, or strengthening exercises deemed adjunct PT necessary by clinical staff. The rate of crossover to receive PT in those studies was similar to the rate of in-person PT use in the early pandemic era analyzed herein. The control group of patients undergoing knee and hip arthroplasties in those studies suggested much higher rates of PT use when left to clinician decision or institutional standards of care alone, where 94.4% of TKA and PKA, and 56.9% of THA patients utilized at least one physical therapy visit post-operatively [19,20]. Our observation that in the later pandemic period PT use did not increase to these levels suggests good patient acceptance of using SDR alone, as other groups also reported that following the lifting of lockdowns, only 53% of patients who used asynchronous rehabilitation at home rejoined in-person PT [12]. It is possible that this could be related to patient fear of infection and desire to avoid healthcare facilities, or in part to lack of access to services at that time. Additionally, the convenience of SDR and its flexibility in allowing patients to complete exercises according to their schedules may contribute to acceptance of this technology without utilizing adjunct PT. Previous reports from this study have shown good compliance with its use, and improved outcomes in those with high compliance [21].

Of interest, we did not observe any differences in either pre-operative or post-operative activity as measured by mean daily steps in the early and late pandemic periods. Previous studies found that patients reported being less active during the early pandemic period when stay-at-home orders were in place [25,26]. However, our data do not indicate that fewer steps were performed by patients in the early pandemic period compared to the later period when restrictions were eased, across procedure types. At 90 days post-operatively, patients in both periods of the pandemic had mean daily step counts that exceeded their pre-operative values, supporting the hypothesis that increased reliance on SDR during the early pandemic phase did not negatively impact recovery of mobility.

Several meta-analyses and systematic reviews have evaluated the effectiveness of telerehabilitation programs in those with musculoskeletal disorders. These larger reviews report that telerehabilitation is effective in providing post-operative exercise and education, where functional outcomes are similar to those achieved by patients utilizing traditional PT following arthroplasty procedures [15,17,18,27,28]. Given the effectiveness of this method, its use has been proposed as being a suitable solution in situations in which in-person PT is not available, as during the COVID-19 pandemic. Few studies assessed the functional outcomes of patients using telerehabilitation during the pandemic; however, Alsobayel and colleagues reported large effects and improved function in those with musculoskeletal disorders utilizing telerehabilitation, though only small portion of the cohort were post-operative patients [29]. Our results indicate similar functional outcomes in patients using SDR throughout the COVID-19 pandemic, where improvements in validated PROMs scores were similar between periods, regardless of the proportion of patients seeking traditional in-person PT. The average change in KOOS JR and HOOS JR scores in this study were similar to those that were reported in RCTs assessing this sbCMP after arthroplasty, where change in KOOS JR was 18.4 points [19] compared to our observation of an increase of 18.2 points. HOOS JR scores increased by 28.4 points in the RCT of sbCMP for THA patients [20], comparable to the 28.4-point improvement demonstrated herein. Importantly, those authors showed that the differences between those prescribed the sbCMP and standard of care did not meet minimally clinical important differences for these PROMs.

Further implementation of telerehabilitation programs such as the one utilized in this study has been advocated by many, particularly since the beginning of the COVID-19 pandemic. The ability of these platforms to produce similar results to those who participate in traditional physical therapy has been associated with many other important benefits. In addition to potentially improving access, use of telerehabilitation has been associated with cost savings of up to $4100 per patient [15]. Moreover, reductions in traditional appointments could have positive environmental impacts by reducing CO_2_ emissions associated with travel [30]. Use of telerehabilitation is expected to reduce the burden of resource utilization on already strained healthcare systems, while providing equivalent care. Landry et al. also point out that as cross-species transmission events such as SARS, MERS, H1N1, and COVID-19 have continued to emerge, the need for telerehabilitation will likely continue to increase in the years to come [13]. Easy to implement solutions, such as the sbCMP used in this study, may bridge the gap in the ability of patients to access traditional physical therapy.

After the end of the public health emergency, use of telehealth services has decreased somewhat, but has continued to persist above pre-pandemic levels [31]. Authors suggest that post-COVID, offering these services should continue; they are easier to implement than during the beginning of the pandemic, as systems are now in place for support [32], and nearly 80% of physicians plan to continue to use telehealth services after the pandemic [33]. The use of a variety of telehealth services has been found to vary amongst populations, where non-white patients, low-income patients, and those with non-private health insurance coverage utilize these services more extensively [31]. Predmore et al. reported that the majority of patients continue to prefer in-person visits for non-emergency needs, though this is somewhat cost-dependent [34]. Telehealth will likely continue to be widely offered, and future research to determine how patient perceptions change over time as this method of care delivery becomes more commonplace is of interest.

This study is strengthened by several factors, including the large sample size and multicenter, prospective nature of the study. However, there are some limitations; the authors did not have access to information regarding the geographical location of each patient which could have impacted access to outpatient services given that restrictions during the pandemic were heterogenous with regards to level and onset throughout the United States, where some states did not enact stay-at-home orders at any time [1]. This is an important limitation, as this may have significantly confounded the results with regard to patients who could no longer access outpatient services. However, with more than 20 participating sites throughout the United States, it is not possible to compare each of the locations to determine whether more stringent lockdowns impacted uptake of the platform. In addition, we relied on patient self-reports of use of adjunct PT collected through the sbCMP which could impact findings. Finally, there may be an element of self-selection bias, as patients who consented to participate may have been more inclined to utilize SDR without adjunct PT. Additional patient selection biases may have been present, as healthier patients may have been preferentially selected to undergo arthroplasty during the pandemic. This study was designed to evaluate the efficacy of a care management platform and assess objective gait quantity and quality metrics, rather than the use of such a platform specifically during the pandemic. Given the goals around gait assessment, populations presenting with comorbidities expected to significantly impact these data (substance abuse or inflammatory arthropathies) were excluded from participation, which could limit generalizability of the findings. Further study in broader populations may be warranted. Finally, some surgeons may require in-person PT in addition to SDR as an institutional standard of care.

In conclusion, our results show that SDR as facilitated by a sbCMP was used without adjunct physical therapy to a greater extent during the early phases of the COVID-19 pandemic without negatively impacting post-operative function. This type of rehabilitation is a feasible and suitable method for the delivery of patient education and guided exercises when patients are unwilling or unable to participate in traditional outpatient physical therapy. Additional research, such as longitudinal studies with longer follow-up and additional patient populations beyond elective unilateral arthroplasty to determine whether broader use self-directed rehabilitation is feasible and effective is warranted.

## Figures and Tables

**Table 1:** Baseline demographics of patients enrolled in the early and late phases of the COVID-19 pandemic separated by procedure type.

	COVID-19 Pandemic Period		
	**Early** (*n* = 551)	**Late** (*n* = 1114)	***p* Value**
**TKA**			
Age (years)	65.1 ± 8.6	64.2 ± 9.0	0.177
BMI (kg/m^2^)	30.9 ± 6.3	31.6 ± 6.4	0.157
Sex (% female)	60.2%	59.0%	0.816
**PKA**			
Age (years)	61.7 ± 8.7	61.8 ± 9.4	0.921
BMI (kg/m^2^)	30.5 ± 5.6	30.4 ± 6.3	0.982
Sex (% female)	53.6%	64.4%	0.192
**THA**			
Age (years)	61.8 ± 10.2	62.1 ± 10.1	0.649
BMI (kg/m^2^)	29.5 ± 6.2	29.0 ± 6.2	0.379
Sex (% female)	56.3%	47.0%	0.024

**Table 2:** Step counts pre-operatively and at 90 days post-operatively as a function of procedure type and period of the pandemic in which procedure occurred.

	COVID-19 Pandemic Period		
	**Early** (*n* = 551)	**Late** (*n* = 1114)	***p* Value**
**TKA**			
Pre	4818.6 ± 3214.1	4655.7 ± 2965.8	0.494
Post	5352.1 ± 3003.1	5164.8 ± 2713.7	0.402
**PKA**			
Pre	5869.1 ± 4370.3	4631.3 ± 3214.4	0.066
Post	6513.6 ± 3533.9	6215.0 ± 3228.7	0.588
**THA**			
Pre	4789.9 ± 3315.8	4826.5 ± 3230.6	0.886
Post	6023.9 ± 3446.6	6093.1 ± 3366.6	0.808

**Table 3:** Changes in PROMS in the early and late periods of the COVID-19 pandemic as measure from pre-operative to 90 days post-operatively.

	Pandemic Period		
	Early	Late	*p* Value
TKA—KOOS JR	17.70	18.36	0.544
PKA—KOOS JR	15.97	16.53	0.801
THA—HOOS JR	28.58	29.00	0.750

## Data Availability

Data presented in this article are not readily available due to commercial restrictions.
